# Divergence in cuticular wax profiles generates partial behavioural isolation between leaf beetle populations with different dispersal traits

**DOI:** 10.1098/rsbl.2025.0105

**Published:** 2025-08-06

**Authors:** Yuki Chiba, Yoshiki Nomura, Masatoshi Hori

**Affiliations:** ^1^ Graduate School of Agricultural Science, Tohoku University, Sendai, Miyagi, Japan

**Keywords:** speciation, behavioural isolation, Chrysomelidae, cuticular wax, sex pheromone, wing polymorphism

## Abstract

Sex pheromones are among the most studied insect mating signals, with their extensive diversity underscoring their crucial role in promoting behavioural isolation during speciation. In Chrysomelidae, cuticular wax (CW), a hydrophobic layer covering the insect cuticle, functions as a mating signal and potentially facilitates behavioural isolation. Male leaf beetles use female CW as a mating signal, and their species-specific profiles prevent heterospecific matings, indicating that divergence in CW profiles may promote reproductive isolation and, hence, contribute to speciation. However, the role of CW as an isolating barrier remains unclear owing to limited knowledge regarding intraspecific divergence in female CW and its coevolution with male preferences. Through chemical analysis and behavioural experiments, we demonstrated that intraspecific divergence in female CW contributes to partial behavioural isolation between leaf beetle populations with different dispersal traits: the flight-capable macropterous and flightless brachypterous forms of *Galerucella grisescens* (Coleoptera: Chrysomelidae). Our results demonstrated a divergence in female CW and male preferences at the intraspecific level, indicating the potential role of CW as an isolating barrier in Chrysomelidae. Additionally, our findings suggest that diverging dispersal ability can enhance divergence in pheromone communication channels, consistent with previous findings that loss of flight enhances beetle diversification.

## 1. Introduction

Behavioural or sexual isolation is a common form of reproductive isolation in sexually reproductive animals [1], and it occurs when mating signals and the receiver’s preference differ between populations. Sex pheromones among insects are extensively well-documented mating signals, and their vast diversity highlights their essential function in generating behavioural isolation during speciation [2].

Cuticular wax (CW) is a potential barrier that generates behavioural isolation among insect populations [3–5]. CW is a hydrophobic layer on the insect cuticle that prevents desiccation stress [6]. Additionally, female CW often functions as sex pheromones, aiding males of various insect species in mate recognition, including Diptera [7–9], Hymenoptera [10], Coleoptera [11–20], Lepidoptera [21,22] and Orthoptera [23]. In Chrysomelidae, males frequently use female CW components as mating signals [13,24–31], and species-specific profiles enable them to distinguish conspecific females from closely related heterospecific females, thereby facilitating reproductive isolation [32–34]. In addition, a laboratory study demonstrated that feeding on different plants induces quantitative changes in CW profiles and affects male mate preferences, indicating an association between behavioural isolation and host shifts [35]. These studies suggest that compositional changes in CW are critical for the speciation process in Chrysomelidae. However, empirical evidence on the role of CW as an isolating barrier is scarce.

**Figure 1.**
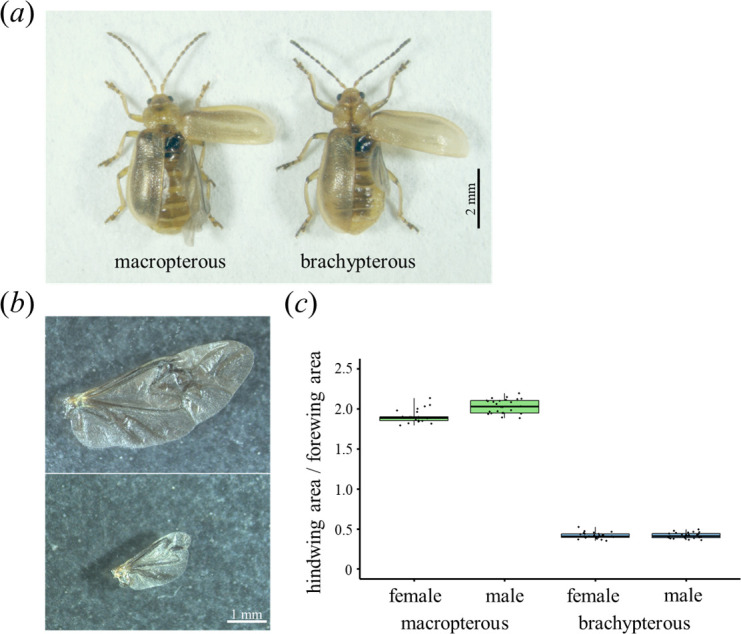
Hindwing polymorphism in *G. grisescens*. (*a*) A macropterous (left) and brachypterous adult (right). (*b*) Hindwings of macropterous (top) and brachypterous forms (bottom). (*c*) Relative hindwing size. The hindwing to forewing area ratio was calculated (*n* = 25).

As various phenotypic differences can accumulate between populations once reproductive isolation ultimately evolves, focusing on conspecific populations in which divergent phenotypes confer partial isolation is effective for identifying traits important for speciation [3,36]. Despite this, genetically based intraspecific divergence in female CW profiles and their coevolution with male preferences have not been reported in Chrysomelidae. Our study aimed to explore the contribution of CW to the speciation of Chrysomelidae. We investigated whether divergence in female CW profiles facilitates behavioural isolation between populations of *Galerucella grisescens* (Coleoptera: Chrysomelidae), in which female CW functions as a sex pheromone [37]. We focused on *G. grisescens* populations with different dispersal traits. *G. grisescens* exhibits genetic variation in hindwing size [38]; macropterous populations can fly with well-developed hindwings, whereas brachypterous populations cannot fly owing to hindwing degeneration (figure 1). Although diverging host-plant preference is essential for promoting speciation in Chrysomelidae [39,40], divergence in dispersal ability may be another crucial evolutionary event for generating behavioural isolation because such a loss of flight ability promotes speciation and contributes to the enormous species richness of Coleoptera [41,42]. Therefore, we hypothesized that divergent CW profiles generate behavioural isolation between macropterous and brachypterous populations of *G. grisescens*. To test this hypothesis, we investigated the CW profiles and male preferences of each population using chemical analyses and mating experiments.

## 2. Methods

### (a) Insects

The macropterous population originated from Ogata, Akita, Japan (40°0ʹ N, 139°56ʹ E), and the brachypterous population originated from Sendai, Miyagi, Japan (38°16ʹ N, 140°52ʹ E). Each population was reared under the same conditions to avoid the potential effects of extrinsic factors (humidity, temperature and nutrition) on CW profiles [43] and to investigate genetically based divergence in phenotypes. Both populations were maintained in a climate chamber at 25 ± 1℃ and 60% relative humidity under a 16 L : 8 D photoperiod and reared on fresh leaves of *Rumex obtusifolius*. After being collected from the fields, both populations were maintained in the laboratory for a minimum of 10 generations on a consistent diet of *R. obtusifolius* fresh leaves and subsequently used in the experiments. Newly emerged adults were sexed and kept separately from each other. Unmated adults at 7–8 days post-emergence were used in the experiments.

### (b) Hindwing polymorphism

To describe hindwing polymorphism, we examined the size of hindwings in females and males from each macropterous and brachypterous population (*n* = 25). After the beetles were freeze-killed, their right forewings and hindwings were dissected. Images were captured using a camera (Axiocam 305 Colour; Zeiss Microscopy, Jena, Germany) on a stereomicroscope (SteREO Discovery.V12, Zeiss Microscopy). Each forewing and hindwing area was measured using ImageJ v. 1.53t [44]. To correct wing size variations caused by body size variations, the size of hindwings was evaluated by calculating the ratios of hindwing areas to forewing areas.

### (c) Cuticular wax extraction

Female and male CW were extracted from 100 individuals of each macropterous and brachypterous population per extraction. After beetles were freeze-killed at −20℃, they were thawed for 15 min at room temperature. They were then immersed in two sequential 20 ml aliquots of *n*-hexane for 30 min each. The two extracts were mixed, and the solvent was evaporated at 39℃ using a rotary vacuum evaporator (N1200A‐S, EYELA, Tokyo, Japan). The extracts were dried under a gentle stream of nitrogen gas. The obtained CW was used for chemical analysis and mating experiments.

### (d) Chemical analysis

To investigate the divergence in the CW profiles, we conducted a chemical analysis. The CW samples were reconstituted in 500 µl of *n*-hexane containing 2 µg of *n*-pentadecane as an internal standard. Three samples were prepared from females and males from each macropterous and brachypterous population. An aliquot of 1 µl of each sample was injected manually into a gas chromatography–mass spectrometry (GC–MS) (GSMS‐QP2010 Ultra, Shimadzu, Kyoto, Japan) system equipped with a DB‐5 ms column (30 m × 0.25 mm i.d., 0.25 µm film thickness, J&W Scientific, CA, USA). The analytical conditions were partly based on those used in previous studies [13,37]. Helium was used as the carrier gas. The inlet temperature was 280℃, and injection was performed in split mode (1 : 10). The oven temperature program was started at 50℃ and heated to 200℃ at a rate of 30℃ min^−1^, followed by an increase to 320℃ at a rate of 2℃ min^−1^ and finally maintained for 15 min. The mass spectrometer was operated in an electron impact mode at 70 eV with a source temperature of 230℃. The major components in the CW of *G. grisescens,* methyl-branched alkanes, were estimated using their mass spectra [45–47] and Kovats indices (KI) [48,49]. The methyl-branched positions were determined based on their characteristic fragment ion peaks [45–47] and differences in their KI values from those of standard *n*-alkanes [49].

### (e) Mating experiment

To elucidate whether male preferences diverged between populations, we conducted mating experiments. All the experiments below were conducted at 25 ± 1℃ during the light period of the rearing condition.

#### (i) Male preference for live females in a two-choice design

First, we explored male mating preferences through two-choice experiments. Macropterous and brachypterous females were released together on a 48 mm diameter glass Petri dish lined with filter paper, after which a male was released in the same test arena. When males attempted aedeagal insertion, we regarded it as ‘chosen’. We recorded which females were first chosen by males (*n* = 60). The experiments were terminated when the males chose either the female or did not attempt aedeagal insertion within 3 h.

#### (ii) Male preference for female cuticular wax

We investigated male preferences for female CW to elucidate whether female CW mediated male mate preferences. A male was released on a 48 mm diameter glass Petri dish lined with filter paper, on which a macropterous and brachypterous female was fixed with double-sided tape on the same centreline passing a circle at the centre of the Petri dish, 1 cm away from the wall. To prevent potential positional bias, the locations of the two females were exchanged for each replicate. The females were subjected to one of three treatments: (i) dead, (ii) reapplied, or (iii) exchanged. This procedure was based on a previous study [37]. Briefly, for treatment 1, live females were freeze-killed at −20℃ for 30 min, following which they were thawed for 15 min at room temperature. For treatment 2, the CW of the freeze-killed females was removed by immersing them in *n*-hexane twice for 30 min each, and then the CW was reapplied by pipetting one female equivalent of the *n*-hexane solution of CW that had been extracted from females of the same wing morph. For treatment 3, *n*-hexane-washed females were covered with one female equivalent of CW extracted from females of different wing morphs. We recorded which females were first chosen by males (*n* = 60). The experiments were terminated when the males chose either the female or did not attempt aedeagal insertion within 3 h.

#### (iii) Male preferences for live females under a no-choice design

When reproductive isolation is well established, mate preferences can be observed under both two- and no-choice designs [50]. Thus, we evaluated male mate preferences in a no-choice situation. A macropterous or brachypterous female was released on a 48 mm diameter glass Petri dish lined with filter paper, followed by a male in the same test arena (*n* = 30). We measured the time taken by males to copulate with paired females. In addition, because *G. grisescens* males inspect potential mates while mounting them and decide whether to attempt subsequent aedeagal insertion [37], we counted the number of times they dismounted from paired females without aedeagal insertion. The experiments were terminated when males copulated with paired females or when 3 h had elapsed since the start of the observations.

### (f) Statistical analysis

The CW profiles between populations were compared by hierarchical clustering analysis using *z*-scores calculated for the peak areas of CW components (peaks occurring at a mean relative abundance of over 0.5% in either or both populations). The analysis was based on the Euclidean distance measure and Ward’s clustering method. Male preferences for live females in the two-choice design were analysed using binomial tests. Trials in which males did not copulate within 3 h were excluded from the statistical analysis. Mate preferences between treatments were compared using Fisher’s exact test with Holm correction to investigate male preferences for female CW. Trials in which males did not copulate within 3 h were excluded from the statistical analysis. The latency to copulate with paired females was compared using log-rank tests when male mate preferences were investigated under a no-choice design. The number of times males dismounted from paired females without copulatory attempts was compared using the Mann–Whitney *U-*test. All analyses were performed using R v. 4.4.1 [51].

## 3. Results

### (a) Hindwing polymorphism

Macropterous individuals possess fully developed hindwings, which are almost twice the size of the forewings and have flying abilities. In contrast, brachypterous individuals are flightless because of the degeneration of the hindwing, which is approximately half the size of the forewings (figure 1).

### (b) Divergence in cuticular wax profile

**Figure 2.**
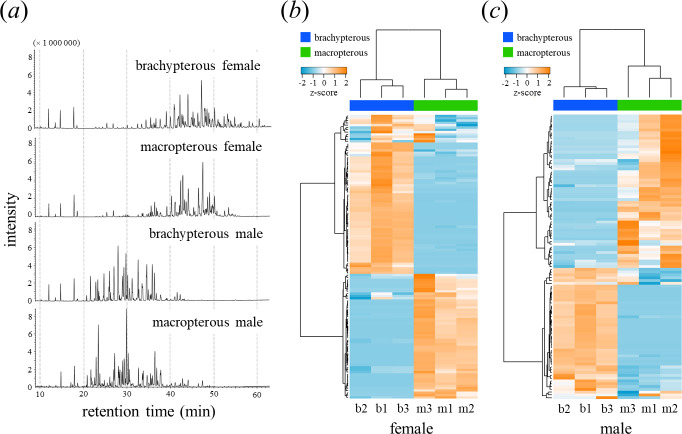
GC–MS analysis of CW profiles. (*a*) Representative total ion chromatograms of CW for brachypterous females, macropterous females, brachypterous males and macropterous males in the respective order from the top. (*b*) Hierarchical clustering analysis using *z*-scores calculated for the abundances (peak areas) of 123 components detected in the female CW. (*c*) Hierarchical clustering analysis using *z*-scores calculated for the abundances (peak areas) of 102 components detected in the male CW. The analysis was based on the Euclidean distance measure and Ward’s clustering method. Each column represents a sample (replicate), and each row represents a compound. b, Brachypterous; m, macropterous; 1–3, samples.

We performed a chemical analysis to determine whether the CW profiles diverged between these populations. In both macropterous and brachypterous populations, CW profiles were qualitatively sexually dimorphic (figure 2*a*), consistent with their roles as sex-discriminating cues [37]. In addition, both female and male CW profiles were qualitatively and quantitatively different between the populations (figure 2; electronic supplementary material, tables S1, S2). Among the 123 components detected from the female CW, 80 components (approximately 65%) were population-specific. Macropterous and brachypterous female CW samples were grouped into distinct clusters (figure 2*b*), indicating that female CW diverged between populations. Among the 102 components detected in male CW, 64 components (approximately 63%) were population-specific. Macropterous and brachypterous male CW samples were grouped into distinct clusters (figure 2*c*), indicating that male CW also diverged between populations.

### (c) Divergence in male preference

**Figure 3.**
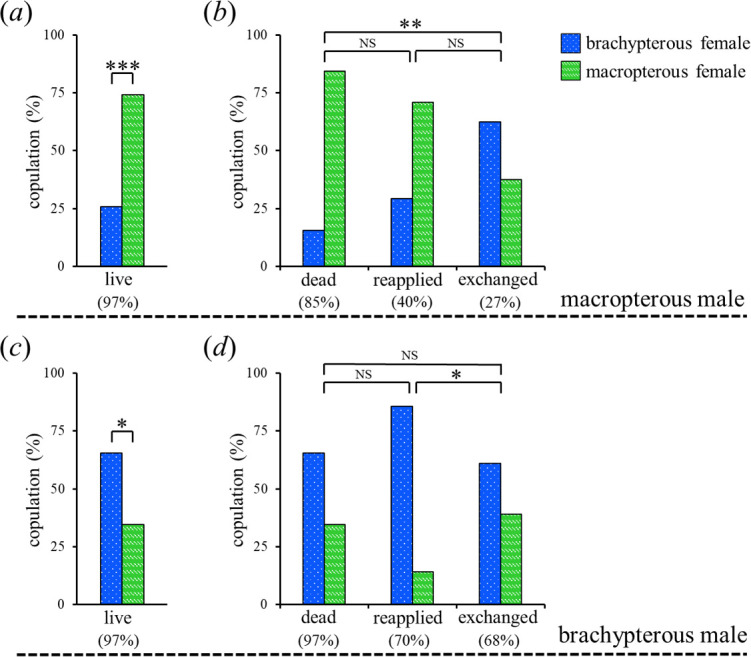
Male mate preferences under the two-choice design. Two live or treated females (macropterous and brachypterous) were presented to a male (*n* = 60). The blue and green bars represent males that chose brachypterous and macropterous females, respectively. (*a*) Preferences for live females in macropterous males. ***p < 0.001, binomial test. (*b*) Preferences for female CW in macropterous males. NS, not significant, **p < 0.01, Fisher’s exact test with Holm correction. (*c*) Preferences for live females in brachypterous males. *p < 0.05, binomial test. (*d*) Preferences for female CW in brachypterous males. NS, not significant, *p < 0.05, Fisher’s exact test with Holm correction. Trials in which males did not copulate within 3 h were excluded from the statistical analysis. Values in parentheses represent percentages of trials in which males copulated with one of the females. Dead: freeze-killed; reapplied: coated with female CW from the same wing morph after being washed with *n*-hexane; exchanged: coated with female CW from the different wing morph after being washed with *n*-hexane.

Two-choice mating experiments showed that live females of the same population were preferred over those from the respective different ones. Macropterous males significantly preferred macropterous females (binomial test, *p* < 0.001; figure 3*a*), and brachypterous males significantly preferred brachypterous females (binomial test, *p* < 0.05; figure 3*c*). Macropterous males preferred the CW profiles of females from their population. Similar to live females, macropterous males preferred macropterous females when given the choice between dead females of the two populations (figure 3*b*). Their preferences for macropterous females were maintained when CW was reapplied and were not significantly different from those of dead females (Fisher’s exact test with Holm correction, *p* > 0.05; figure 3*b*). However, their preferences were reversed when the female CW was exchanged, where a greater percentage of macropterous males chose brachypterous females coated with macropterous female CW over macropterous females coated with brachypterous female CW, and the male mate preferences were significantly different from those towards dead females (Fisher’s exact test with Holm correction, *p* < 0.01; figure 3*b*). However, the mating responses of macropterous males towards reapplied and exchanged females decreased (40 and 27%, respectively), and no significant difference was observed between them (Fisher’s exact test with Holm correction, *p* > 0.05; figure 3*b*). Brachypterous males did not show an apparent preference for the brachypterous female CW. They preferred brachypterous females when dead females were present (figure 3*d*). Their mating preference was not reversed when the female CW was reapplied or exchanged, with no significant differences from dead females (Fisher’s exact test with Holm correction, *p* > 0.05; figure 3*d*); however, a significant difference was observed between reapplied and exchanged females (Fisher’s exact test with Holm correction, *p* < 0.05; figure 3*d*). In the no-choice design, males copulated with females from both populations. Macropterous males exhibited no mate preferences under the no-choice design, as no significant differences were observed in the latency to copulation (log-rank test, *p* > 0.05; electronic supplementary material, figure S3a) and the number of times that they ended their mating attempt without aedeagal insertion (Mann–Whitney *U-*test, *p* > 0.05; electronic supplementary material, figure S3b) between the two morphs of females. In contrast, brachypterous males preferred brachypterous females in the no-choice design. Compared with brachypterous females, brachypterous males showed significantly delayed copulation with macropterous females (log-rank test, *p* < 0.05; electronic supplementary material, figure S3c). Furthermore, they often dismounted from macropterous females without aedeagal insertion, and the frequency of this behavioural pattern was significantly higher than that towards brachypterous females (Mann–Whitney *U* test, *p* < 0.001; electronic supplementary material, figure S3d).

## 4. Discussion

Although previous findings indicate that the CW is fundamental in the speciation process of Chrysomelidae, empirical evidence remains limited. Our study showed that intraspecific divergence in female CW profiles contributed to partial behavioural isolation between two *G. grisescens* populations with different dispersal abilities. These results support the hypothesis that divergence in CW profiles may generate behavioural isolation and may be associated with the speciation process in Chrysomelidae. Additionally, although host preference has a fundamental role in the speciation of Chrysomelidae [39,40], our study suggests that flight loss may also be an evolutionary event leading to behavioural isolation.

Our study identified divergence in female sex pheromones, which comes along with divergence in male mating preferences. However, the evolution of the pheromone communication channels remains unclear. The conventional perspective is that sex pheromones are under stabilizing sexual selection, with highly specific male preferences resulting in a lack of variation in female pheromones. Thus, the evolution of sex pheromones appears to be difficult [2]. However, stabilizing selection cannot explain the enormous diversity of insect pheromones, suggesting alternative evolutionary forces [52]. Selective pressures on pheromones resulting from abiotic and biotic factors can be evolutionary forces driving signallers–receivers coevolution [5,53,54]. CW functions as physical barriers to desiccation as well as pheromones [6,43,55]. Therefore, CW profiles evolve with adaptation to abiotic environmental factors, such as temperature and humidity [5,56], which has also been confirmed by experimental evolution [57]. Additionally, CW is potentially under selective pressure from biotic factors [58–60] because some predatory insects, parasitoids and parasites use CW as prey and host recognition cues [61–66]. Based on these insights and the potential ecological isolation between macropterous and brachypterous forms in *G. grisescens* [38], adaptation to contrasting environments may lead to divergence in CW profiles. Furthermore, females with altered CW profiles could have higher fitness, and selective pressure might favour male preferences for these evolved female CW, driving behavioural isolation. Consistent with our assumption, the contribution of CW differentiation to the speciation process has been reported in some non-coleopteran taxa [67–69].

We showed that partial behavioural isolation evolved between two allopatric populations of *G. grisescens* with different dispersal abilities, consistent with previous findings that the loss of flight ability promotes allopatric speciation in Coleoptera [41,42]. In certain regions, macropterous and brachypterous forms are found sympatrically [38]. Notably, despite the sympatry, the frequency of intermediate morphs was low [38], suggesting that some forms of pre- and/or post-mating isolation exist between sympatric macropterous and brachypterous morphs. Insects, including leaf beetles, may exhibit accelerated mate preferences between sympatric populations compared with that of allopatric populations owing to selection against producing less-fit hybrids, a process known as reinforcement [70–73]. Thus, if hybrids between macropterous and brachypterous morphs exhibit reduced fitness, the divergence in CW and male preferences between sympatric populations should be even more pronounced than that observed in allopatric populations. Further studies are required to investigate behavioural isolation between sympatric populations.

Although males evolved their preferences for females from their populations, they copulated with both macropterous and brachypterous females in no-choice situations, suggesting that they were still able to recognize both females as potential mates, possibly because these populations were currently in an early stage of divergence. As brachypterous males did not show an apparent preference for CW from brachypterous females, they possibly relied on CW components common to both macropterous and brachypterous females. Additionally, other contact signals, such as polar contact chemicals that were not extracted with *n*-hexane or physical cues detected by direct touch, may significantly influence their mate preferences, as they hesitated to copulate with macropterous females following direct tactile interaction. However, in addition to common CW components, macropterous males likely use macropterous-specific chemicals for mate recognition, as they prefer macropterous female CW profiles. However, similar to brachypterous males, macropterous males also potentially use additional synergistic mating cues because their mating preference towards exchanged females was not completely reversed when compared with the case involving live and dead females. To comprehensively understand their communication systems, further studies are needed. Furthermore, elucidating the reasons for the decline in the mating responses of macropterous males towards reapplied and exchanged females is necessary. Submerging the beetles in *n*-hexane for a relatively long time (30 min) might have resulted in the extraction of non-CW compounds, which affected male responses. Additionally, the reapplication of CW might have altered the original structure of CW, such as nanoscale wax layers [74] and heterogeneous distribution patterns of wax chemicals [75,76], negatively affecting male responses.

## 5. Conclusion

Our study demonstrated that divergence in CW profiles contributes to partial behavioural isolation between *G. grisescens* populations with different dispersal traits, supporting the hypothesis that CW may act as an isolating barrier in the speciation process of Chrysomelidae. Furthermore, our findings suggest that diverging dispersal ability can lead to the evolution of sex pheromone communication channels, which is consistent with previous findings that flight loss enhances beetle diversification.

## Data Availability

Data and code are available online from the Dryad Digital Repository [77]. Supplementary material is available online [78].
